# Imaging of cerebrovascular pathology in animal models of Alzheimer's disease

**DOI:** 10.3389/fnagi.2014.00032

**Published:** 2014-03-13

**Authors:** Jan Klohs, Markus Rudin, Derya R. Shimshek, Nicolau Beckmann

**Affiliations:** ^1^Institute for Biomedical Engineering, University of Zurich and ETH ZurichZurich, Switzerland; ^2^Neuroscience Center Zurich, University of Zurich and ETH ZurichZurich, Switzerland; ^3^Institute of Pharmacology and Toxicology, University of ZurichZurich, Switzerland; ^4^Autoimmunity, Transplantation and Inflammation/Neuroinflammation Department, Novartis Institutes for BioMedical ResearchBasel, Switzerland; ^5^Analytical Sciences and Imaging, Novartis Institutes for BioMedical ResearchBasel, Switzerland

**Keywords:** Alzheimer's disease (AD), amyloid-beta (Aβ), angiography, cerebral amyloid angiopathy (CAA), cerebral blood flow (CBF), magnetic resonance imaging (MRI), microscopy, transgenic mice

## Abstract

In Alzheimer's disease (AD), vascular pathology may interact with neurodegeneration and thus aggravate cognitive decline. As the relationship between these two processes is poorly understood, research has been increasingly focused on understanding the link between cerebrovascular alterations and AD. This has at last been spurred by the engineering of transgenic animals, which display pathological features of AD and develop cerebral amyloid angiopathy to various degrees. Transgenic models are versatile for investigating the role of amyloid deposition and vascular dysfunction, and for evaluating novel therapeutic concepts. In addition, research has benefited from the development of novel imaging techniques, which are capable of characterizing vascular pathology *in vivo*. They provide vascular structural read-outs and have the ability to assess the functional consequences of vascular dysfunction as well as to visualize and monitor the molecular processes underlying these pathological alterations. This article focusses on recent *in vivo* small animal imaging studies addressing vascular aspects related to AD. With the technical advances of imaging modalities such as magnetic resonance, nuclear and microscopic imaging, molecular, functional and structural information related to vascular pathology can now be visualized *in vivo* in small rodents. Imaging vascular and parenchymal amyloid-β (Aβ) deposition as well as Aβ transport pathways have been shown to be useful to characterize their dynamics and to elucidate their role in the development of cerebral amyloid angiopathy and AD. Structural and functional imaging read-outs have been employed to describe the deleterious affects of Aβ on vessel morphology, hemodynamics and vascular integrity. More recent imaging studies have also addressed how inflammatory processes partake in the pathogenesis of the disease. Moreover, imaging can be pivotal in the search for novel therapies targeting the vasculature.

## Introduction to cerebrovascular pathology in alzheimer's disease

Alzheimer's disease (AD) is the most common form of dementia in elderly individuals. The disease has been classically viewed as the accumulation of amyloid-β (Aβ), generated by proteolytic cleavage of the amyloid precursor protein (APP), in the brain parenchyma (Aβ plaques), leading to Aβ-related neuropathology and loss of cognitive function (Hardy and Selkoe, 2002). Increasing evidence has implicated cerebrovascular dysfunction in the etiology of the disease (for reviews see Thal et al., 2008a,b; Bell and Zlokovic, 2009; Weller et al., 2009; Biffi and Greenberg, 2011). Epidemiological studies indicate a strong overlap between AD pathology and cardiovascular disease, suggesting that they might share common mechanisms and risk factors. Among all cerebrovascular comorbidities in AD, cerebral amyloid angiopathy (CAA) is the most common pathological finding, present in up to 90% of AD patients (Vinters, 1987; Jellinger, 2002). CAA results from the failure to eliminate Aβ from the cerebral vasculature (Weller et al., 2009). Both AD and CAA can lead to pronounced cerebrovascular dysfunction, characterized by impaired neurovascular and metabolic regulation of cerebral blood flow (CBF) and aberrations in vascular morphology and density. In addition, changes in the proteolytic microenvironment and inflammation lead to impairment of blood-brain barrier (BBB) integrity and the occurrence of cerebral microbleeds (CMBs) and intracerebral hemorrhages (Snowdon, 2003; Cordonnier and van der Flier, 2011). It has been suggested that the vascular pathology may mutually interact with neurodegeneration in AD, and thus aggravate cognitive decline, though the relationship between these two processes is poorly understood. Hence, recent research has been increasingly focused on understanding the link between cerebrovascular alterations and AD. This has on one hand been spurred by the engineering of transgenic animals, which display pathological features of AD and develop CAA to various degrees. These models have been proven versatile for elucidating the role of amyloid deposition, vascular dysfunction and for evaluating novel therapeutic concepts. On the other hand, research has benefited from the development of novel imaging techniques, which are capable of characterizing vascular pathology *in vivo*. They provide vascular structural read-outs and have the ability to assess the functional consequences of vascular dysfunction as well as to visualize and monitor the molecular processes underlying these pathological alterations.

This article focusses on recent *in vivo* small animal imaging studies addressing vascular aspects related to AD. We first introduce transgenic mouse models of AD displaying CAA and their main characteristics, followed by a summary of the current imaging techniques and discuss their advantages and limitations. The potential of imaging vascular pathology will be illustrated by discussing applications on the visualization of vascular amyloid deposition and amyloid clearance pathways, the assessment of the cerebrovascular architecture to elucidate the dynamics and mechanism of CAA and to understand how amyloid deposition induces vascular remodeling. The use of functional imaging read-outs to monitor the deleterious consequences of amyloid deposition, namely chronic hypoperfusion and reduced hemodynamic response are presented. The role of neurovascular inflammation, loss of BBB as well as CMBs in advanced stages of the disease are then addressed. The fact that CAA may be halted or even reversed is evaluated by a glance on therapeutic studies involving the animal models. Finally, we evaluated how biological lessons learned from these models may be translated into the clinic.

## Transgenic models of alzheimer's disease displaying cerebral amyloid angiopathy

To date, several mouse and rat lines have been genetically engineered to serve as preclinical models for AD. Most of these models have been generated by transgenic overexpression of the gene encoding for the human APP, which leads to progressive accumulation of Aβ and amyloidosis in the brain. Some of these strains develop CAA to various degrees, which allow studying the effect of Aβ accumulation on vascular function. Further strains have been established addressing the other pathological hallmark of AD, neurofibrillary tangles, by expressing different forms of tau (Gotz et al., 1995; Duff et al., 2000; Allen et al., 2002; SantaCruz et al., 2005). Furthermore, mouse models with multiple mutations have also been engineered, e.g., APP/PS1 and 3xTg-AD, to study either the enhancement of amyloid pathology by presenilin mutations or the interaction between amyloid and tau (Blanchard et al., 2003; Oddo et al., 2003a,b). However, most studies utilize strains with a single mutation, which have the advantage of assessing one pathological process at a time without being confounded by the complex pathophysiology of sporadic AD. Transgenic mouse models are very valuable for drug discovery as the pathology usually develops within months (as compared to years or decades in AD patients) and disease pathological stages are well characterized.

Mice overexpressing APP have been utilized as valid models for CAA (summarized in Table 1). These transgenic models use various promoters to drive transgene expression in different genetic backgrounds. Interestingly, it has been shown that neuronal Aβ is the driver for CAA (Calhoun et al., 1999) and an impaired Aβ clearance seems to enhance CAA (Herzig et al., 2004). Early onset CAA is observed in models with multiple autosomal dominant mutations like Thy1-APP751, Tg-SwDI, TgCRND8, whereas late-onset CAA (>9 months of age) is usually detected in mice with expression of mutated APP restricted to one familiar mutation as in Tg2576, PDAPP, APPDutch, APP/London, APP23, or TgAPPArc animals. CAA is observed earlier in mice that additionally carry a presenilin mutation like APPswe/PS1dE9 or Thy1-APP751SLxHMG-PS1M146L.

**Table 1:** **Summary of transgenic models used in AD research and their relation to CAA development**.

**Line (Alternative designation)**	**Construct Promoter**	**Familial APP AD mutation**	**Onset of amyloid plaques**	**CAA onset**	**CAA severity**	**Neuronal loss**	**Cognitive defects**	**References**
**APP TRANSGENIC MOUSE MODELS**								
Tg2576 (APPsw)	hAPP695	Swe	7–10 mo	9–12 mo	++	No	Yes	Hsiao et al., 1996; Fryer et al., 2003; Domnitz et al., 2005; Kumar-Singh et al., 2005; Perez-Cruz et al., 2011
	K670N/M671L							
	HamPrP							
Thy1-APP751 (TASD-41, mThy1-hAβ PP751)	hAPP751	Swe	3–4 mo	5–7 mo	+	No	Yes	Rockenstein et al., 2001; Havas et al., 2011
	K670M/N671L/V717I	Lon						
	mThy1							
ArcAbeta (ArcAβ)	hAPP695 K670N/M671L/E693G	Swe	5–7 mo	9–15 mo	+++	No	Yes	Knobloch et al., 2007; Klohs et al., 2012
	MoPrP	Arc						
Tg-SwDI (APPSwDI)	hAPP770	Swe	3 mo	6 mo	+++	No	Yes	Davis et al., 2004; Miao et al., 2005; Xu et al., 2007
	K670N/M671L/E693Q/D694N	Dut						
	mThy1	Iow						
TgCRND8	hAPP695 KM670/671NL/V717F	Swe	3 mo	6–7 mo	++	No	Yes	Chishti et al., 2001; Domnitz et al., 2005; Lovasic et al., 2005
	HamPrP	Ind						
PDAPP	hAPP full-length	Ind	6–9 mo	10–12 mo	+	n.a.	Yes	Games et al., 1995; Dodart et al., 1999; Chen et al., 2000; Fryer et al., 2003; Nilsson et al., 2004; Domnitz et al., 2005; Hartman et al., 2005; Daumas et al., 2008; Schroeter et al., 2008
	V717F							
	PDGFb							
APPDutch	hAPP	Dut	–	22–25 mo	++	No	n.a.	Herzig et al., 2004, 2007
	E693Q							
	mThy1							
APP/London (APP/Lo, APP(V717I), APP-Ld, APP/Ld, APP/V717I, APP[V717I])	hAPP695	Lon	10–11 mo	12–15 mo	++	No	Yes	Moechars et al., 1999; Dewachter et al., 2000; van Dorpe et al., 2000; Tanghe et al., 2010; Perez-Cruz et al., 2011
	V717I							
	mThy1							
BRI-Aβ 42	BRI-Ab42 fusion		3 mo	12 mo	++	n.a.	n.a.	McGowan et al., 2005
	MoPrP							
APPArcSwe (tg-APP(ArcSwe), TgArcSwe)	hAPP	Swe	5–6 mo	9 mo	+	n.a.	n.a.	Lord et al., 2006
	KM670/671NL/E693G	Arc						
	mThy1							
J20 hAPP	hAPP	Swe	5–7 mo	<11 mo	+	Yes	Yes	Mucke et al., 2000; Spilman et al., 2010; Thanopoulou et al., 2010; Wright et al., 2013
	K670N/M671L/V717F	Ind						
	PDGFb							
APP23	hAPP751	Swe	6 mo	12 mo	++	Yes	Yes	Sturchler-Pierrat et al., 1997; Calhoun et al., 1998, 1999; Winkler et al., 2001
	K670N/M671L							
	mThy1							
TGAPParc	hAPP695	Arc	9 mo	<18 mo	+	n.a.	Yes	Rönnbäck et al., 2011, 2012
	E693G							
	mThy1							
APP23	hAPP751	Swe	n.a.	n.a.	+++	n.a.	n.a.	Herzig et al., 2009
X	K670N/M671L	Dut						
APPDutch	X							
	E693Q							
	mThy1							
E22ΔAβ	hAPP695	Swe	–	<24 mo	++	n.a.	Yes	Kulic et al., 2012
	K670N/M671L/E693Δ	Osaka						
	MoPrP							
tTA/APP (APP/TTA)	m/hAPP695	Swe	6 mo	n.a.	n.a.	n.a.	Yes	Jankowsky et al., 2005; Melnikova et al., 2013
	K570M/N571L/V617F	Ind						
	tet							
**APPxPS TRANSGENIC MOUSE MODELS**								
APPswe/PS1dE9 (APP/PS1)	m/hAPP695 K595N/M596L	Swe	6–7 mo	6 mo	++	n.a.	Yes	Jankowsky et al., 2001; Savonenko et al., 2005; Garcia-Alloza et al., 2006; O'Leary and Brown, 2009; Stover and Brown, 2012
	X							
	hPS1							
	dE9							
	MoPrP							
	coinjection							
Tg2576	hAPP695	Swe	3–6 mo	10 mo	++	Yes	Yes	Holcomb et al., 1998; Sadowski et al., 2004; Kumar-Singh et al., 2005; Wang et al., 2012a
X	K670M/N671L							
PS1 M146L	X							
(APP/PS1, TgPSAPP, PSAPP)	PS1							
	M146L							
	HamPrP							
Thy1-APP751SL	hAPP751	Swe	3–5 mo	3–5 mo	++	Yes	n.a.	Blanchard et al., 2003; Schmitz et al., 2004; El Tayara et al., 2010
X	K670M/N671L/V717I	Lon						
HMG-PS1M146L	mThy1							
(APPSweLon/PS1M146L, APP/PS1)	X							
	PS1							
	M146L							
	HMG							
APPDutch	hAPP	Swe	3 mo	n.a.	+	n.a.	n.a.	Herzig et al., 2004
X	E693Q	Dut						
PS45	X							
	PS1							
	G384A							
	mThy1							
APP23	hAPP751	Swe	2–3 mo	n.a.	+	n.a.	Yes	Busche et al., 2008, 2012; Beckmann et al., 2011
X	K670N/M671L							
PS45	X							
	PS1							
	G384A							
	mThy1							
PS2APP (PS2_N141I_XAPP_Swe_)	hAPP751	Swe	5 mo	12 mo	+	n.a.	Yes	Richards et al., 2003; Woolley and Ballard, 2005; Weidensteiner et al., 2009
	K670N/M671L							
	mThy1							
	X							
	PS2							
	N141I							
	MoPrP							
APPPS1 (APPPS1-21)	hAPP751	Swe	2–4 mo	8 mo	+	Yes	Yes	Radde et al., 2006; Rupp et al., 2011; Montagne et al., 2012; Vom Berg et al., 2012
	K670N/M671L/V717I							
	mThy1							
	X							
	PS1							
	L166P							
	knock-in							
APPxPS1-Ki (APP^SL^PS1KI)	hAPP751	Swe	2–3 mo	n.a.	n.a.	Yes	Yes	Casas et al., 2004; Faure et al., 2011
	K670N/N671L	Lon						
	mThy1							
	PS1							
	M233T/L235P							
	mThy1							
	coinjection							
5XFAD	hAPP695	Swe	2–3 mo	n.a.	n.a.	Yes	Yes	Oakley et al., 2006; Kimura and Ohno, 2009; Jawhara et al., 2012
	K670N/M671L/I716V/V717I	Lon						
	mThy1	Flo						
	PS1							
	M146L/L28V							
	mThy1							
	coinjection							
**APPxPSXTAU TRANSGENIC MOUSE MODELS**								
3xTg-AD (3xTg)	hAPP695	Swe	12–15 mo (heterozy-gous)	n.a.	n.a.	Yes.	Yes	Oddo et al., 2003a,b; Billings et al., 2005; Bittner et al., 2010
	K670M,N671L + htau (P301L)							
	mThy1							
	coinjection		6 mo (homozy-gous)					
	PS1 (M146V)							
	knock-in							
**APP DOUBLE TRANSGENIC RAT MODELS**								
TgF344-AD	hAPP695 K595N/M596L + hPS1(dE9)	Swe	6 mo	6–12 mo	++	Yes	Yes	Cohen et al., 2013
	MoPrP							
	coinjection							

*Mutations: Arctic (Arc), Dutch (Dut), Florida (Flo), Indiana (Ind), London (Lon), Swedish (Swe). Classification of CAA severity: ^+^ mild, ^++^ significant, ^+++^ pronounced. Abbreviations: n.a., not analysed/not available; mo, months; X: cross breeding*.

Experimental evidence suggests that total levels of Aβ as well as the ratio of the Aβ_40_ and Aβ_42_ peptides (Aβ_40_/Aβ_42_), generated by proteolytic cleavage of β- and γ-secretase, are factors determining both onset and the severity of CAA (Herzig et al., 2004, 2007; for reviews see Herzig et al., 2006; Kumar-Singh, 2009). APPDutch animals displaying a very high ratio of Aβ_40_/Aβ_42_ develop severe CAA (Herzig et al., 2004), indicating that the majority of Aβ deposited in vascular deposits is Aβ_40_. Furthermore, APP23xAPPDutch double transgenic animals with an overall increase of Aβ load and a high ratio Aβ_40_/Aβ_42_ show enhanced CAA compared to APP23 single transgenic mice. In contrast, APPDutchxPS45 with a lower Aβ_40_/Aβ_42_ ratio develop more pronounced parenchymal than vascular deposits (Herzig et al., 2004). Also, autosomal dominant mutations with reduced Aβ_40_/Aβ_42_ ratio such as the Indiana mutation in PDAPP and APP/London mice display less pronounced CAA. These observations are in line with findings in hereditary cerebral hemorrhage patients with amyloidosis of the Dutch type. Individuals with this rare autosomal dominant disorder, caused by an APP 693 mutation that leads to recurrent hemorrhagic strokes and dementia, have decreased Aβ_42_ levels in the brain (Bornebroek et al., 2003). However, contrasting data have been reported for the BRI-Aβ_42_ animals, where overexpression solely of Aβ_42_ led to CAA, while overexpression of Aβ_40_ did not (McGowan et al., 2005). Other reports suggested that Aβ_40_ can inhibit fibril formation and even inhibit amyloid deposition (Jarrett et al., 1993; Kim et al., 2007). Taken together, most studies indicate that Aβ_42_ might be essential for the initial amyloid deposition in vessels and that an increase of overall total Aβ as well as of the Aβ_40_/Aβ_42_ ratio favors subsequent vascular Aβ load.

In this review, we focus on effects of Aβ on the cerebral vasculature and the consequences thereof. Since the transgenic AD models exhibit quite different levels of CAA vs. neuritic Aβ deposits, they enable studying the effect of these processes on AD pathology.

## Imaging modalities for the characterization of cerebral vasculature

**Figure 1:**
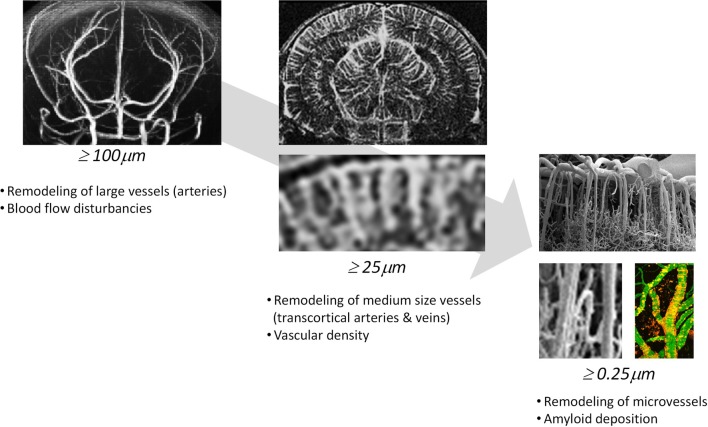
**Phenotypic characterization of cerebrovascular structures at various length scales**. Time-of-flight magnetic resonance angiography can depict large vessels (≥100μ*m*), contrast-enhanced magnetic resonance angiography can depict medium sized vessels (≥50μ*m*) and two-photon microscopy can visualize microvessels (≥0.25μ*m*).

Multimodal imaging offers an impressive number of approaches for characterizing cerebral vasculature from the cellular to the whole organ scale (Table 2). The dimensions of cerebral vessels span a range of 2–3 orders of magnitude with large arteries and veins of dimensions of approximately 1 mm to capillaries with typical diameters of 5–10 μm. Correspondingly, the phenotypic characterization of cerebral vasculature under normal and pathological conditions requires information at multiple length and time scales addressing various aspects of vascular anatomy and function/physiology (Figure 1).

**Table 2:**
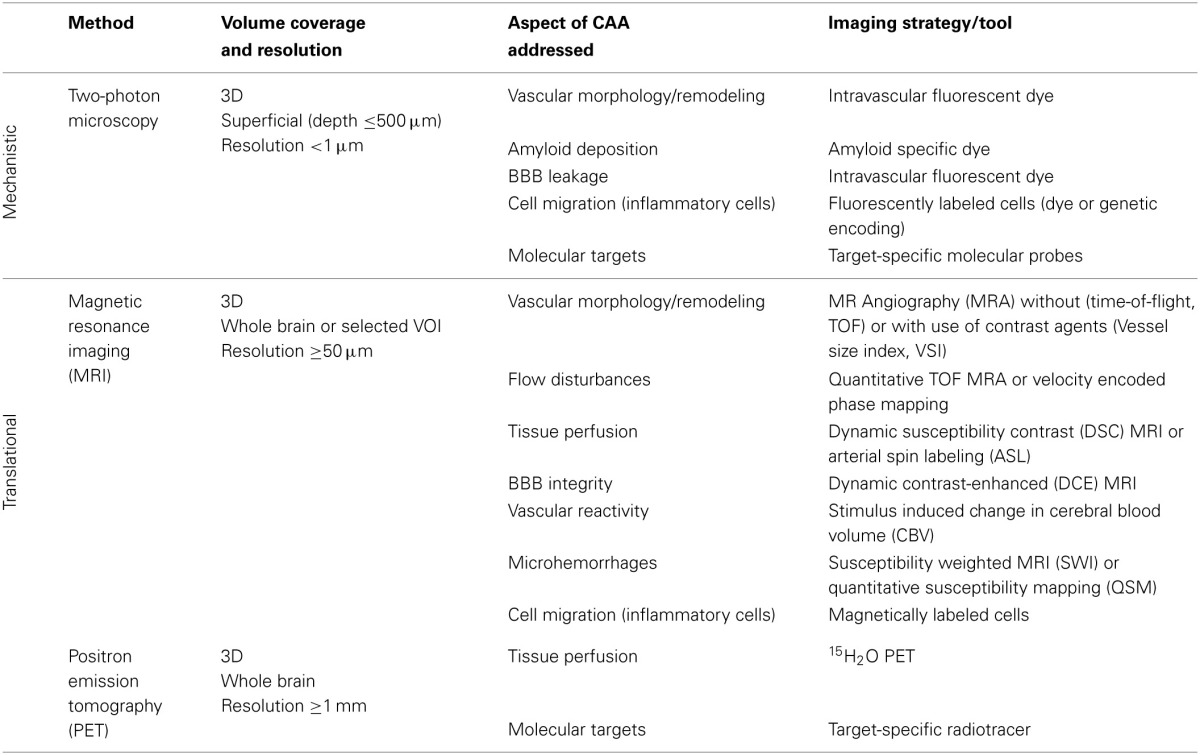
***In vivo* imaging techniques applied to AD models**.

Light-based microscopy methods such as two-photon microscopy or optical coherence tomography provide exquisite information at submicrometer resolution though they are limited to superficial structures due to light scattering by turbid tissue. The cortex is ideally suited for *in vivo* microscopy of adult mice. Technically, it involves the preparation of an optical window in anesthetized animals, comprising either a thinned skull region or a sealed craniotomy. For short- and long-term imaging experiments the thinned skull preparation is the preferred method because it is the least invasive to parenchymal tissue (Helm et al., 2009). Recording duration ranges from minutes to months depending on the biological process investigated. Some applications, for instance high-resolution two-photon imaging of extensive cortical areas or micrometerscale structures deep inside the cortex (~250 μm), require the use of an open skull window providing direct access to the brain parenchyma (Holtmaat et al., 2009). To target deeper brain structures like the hippocampus, removal of parts of the cortex have been proven to be feasible (Busche et al., 2012). It should be kept in mind that skull removal may lead to mechanical injuries to the cortical surface or immediate disturbances in local blood perfusion, BBB permeability, and brain homeostasis, while removal of whole brain structures might even lead to damage of brain structures.

Modalities such as computed tomography (CT), magnetic resonance imaging (MRI), positron emission tomography (PET), single-photon emission computed tomography (SPECT) and near-infrared fluorescence (NIRF) imaging allow for non-invasive three-dimensional (3D) coverage of large volumes at the expense of spatial resolution. Imaging solutions based on these technologies are potentially translatable. Due to its versatility, MRI has been extensively used for characterizing cerebral vasculature: gross vascular architecture, tissue perfusion, integrity of the BBB, occurrence of hemorrhages and immune cell infiltration, all have been studied both in patients and animal models of the disease. These structural and functional read-outs can be complemented by molecular information derived from the use of target specific probes (Klohs and Rudin, 2011).

A brief summary of imaging activities and techniques related to murine AD models is provided in Table 3. A glance at Tables 2, 3 reveals that both macroscopic techniques, like MRI and PET, as well as *in vivo* microscopy are at the center of attention. The method of choice depends on the required resolution of the method, and thus on the specific research questions addressed.

**Table 3:** ***In vivo* imaging activities and techniques related to AD models**.

**Line**	**Imaging activities and techniques**	**References**
Tg2576 (APPsw)	High-resolution T2^*^-weighted magnetic resonance microscopy	Luo et al., 2010
	Metabolism and function (FDG-PET and CBV fMRI)	Luo et al., 2012
	Microhemorrhages (antibody-coated iron oxide nanoparticles, MRI)	Poduslo et al., 2011
	Vascular structure (MRA)	Kara et al., 2012
	ROS and MMP activity, CAA (multiphoton microscopy)	Garcia-Alloza et al., 2009; Gregory et al., 2012
	Resorufin analogs for CAA detection (PET)	Han et al., 2011
	Macromolecular changes (magnetization transfer contrast MRI)	Perez-Torres et al., 2014
	Changes in water diffusion (diffusion tensor imaging, DTI)	Sun et al., 2005
	Development of Aβ plaques (MRI)	Braakman et al., 2006
	Axonal transport rates (manganese-enhanced MRI, MEMRI)	Smith et al., 2007; Wang et al., 2012c
	Plaque formation, astrocytic Ca^2+^ signaling (long-term two-photon* in vivo* imaging)	Takano et al., 2007; Burgold et al., 2011
	Plaque detection ([11C]-PIB PET)	Snellman et al., 2013
	CAA formation (multiphoton microscopy)	Robbins et al., 2006
	Vasomotor dysfunction (Laser-Doppler flowmetry)	Park et al., 2013
	Axonal transport, blood flow (manganese-enhanced MRI, rCBF)	Massaad et al., 2010
ArcAbeta	Microhemorrhages (quantitative susceptibility mapping QSM, CE-MRA)	Klohs et al., 2011, 2012
	Function (CBV-MRI) BBB Klohs et al., 2013	Princz-Kranz et al., 2010
Tg-SwDI (APPSwDI)	Astrocytic Ca^2+^ signaling (two-photon *in vivo* imaging)	Takano et al., 2007
	Vasomotor dysfunction (Laser-Doppler flowmetry)	Park et al., 2013
TgCRND8	Astrocyte detection (bioluminescence)	Watts et al., 2011
	Amyloid imaging (6E10-PEG, PET)	McLean et al., 2013
	Microvascular structure (*in viv*o two-photon laser scanning microscopy)	Dorr et al., 2012
PDAPP	White matter injury (DTI)	Song et al., 2004
	Brain volumetric changes (MRI volumetry)	Redwine et al., 2003
	Hippocampal volume (MRI volumetry)	Weiss et al., 2002
	Blood volume (CBV fMRI)	Wu et al., 2004
	Inflammation (multiphoton microscopy)	Koenigsknecht-Talboo et al., 2008
APP/London (APP(V717I))	Hypointense brain inclusions (MRI)	Vanhoutte et al., 2005
J20 hAPP	perfusion (ASL)	Hébert et al., 2013
APP23	Vascular changes (MRA, fMRI)	Mueggler et al., 2002, 2003; Beckmann et al., 2003, 2011; Krucker et al., 2004; Thal et al., 2009
	Plaque and glia detection (PET, bioluminescence, fluorescence molecular tomography–computerized tomography, NIRF imaging)	Okamura et al., 2004; Hintersteiner et al., 2005; Higuchi, 2009; Hyde et al., 2009; Watts et al., 2011; Snellman et al., 2013
	Vascular changes (vascular corrosion casting and scanning electron microscopy)	Meyer et al., 2008
	Neuroinflammation, glia detection (PET)	Maeda et al., 2007, 2011
APPswe/PS1dE9 (APP/PS1)	Blood volume, parenchymal and vascular deposits (MRI, rCBV, CBF)	Hooijmans et al., 2007a,b
	ROS and MMP activity and plaque detection (multiphoton microscopy)	Garcia-Alloza et al., 2009; Nabuurs et al., 2012
	Plaque detection ([11C]-C-PIB, PET)	Snellman et al., 2013
	Neurovascular coupling (optical-resolution photoacoustic microscopy)	Hu et al., 2009
	Microglia imaging (PET)	Venneti et al., 2009
Tg2576	Deformation-based morphometry (3D MRI) and metabolite concentration (^1^H MR spectroscopy)	Oberg et al., 2008
X		
PS1 M146L (APP/PS1, TgPSAPP, PSAPP)		
	Morphological changes (deformation-based morphometry)	Lau et al., 2008
Thy1-APP751SL	MR relaxation times and vascular changes (MRA)	El Tayara Nel et al., 2007; El Tayara et al., 2010
X		
HMG-PS1M146L (APPSweLon/PS1M146L, APP/PS1)	Metabolism and function (FDG-PET)	Poisnel et al., 2012
APP23	*In vivo* Ca^2+^ imaging (two-photon microscopy)	Busche et al., 2008, 2012; Grienberger et al., 2012
X		
PS45		
3xTg-AD (3xTg)	White matter pathology (anatomical MRI and DTI)	Bittner et al., 2010; Fuhrmann et al., 2010; Kastyak-Ibrahim et al., 2013
	Dendritic spine loss (*in vivo* two-photon and confocal imaging)	
	Inflammation (two-photon microscopy)	
TgF344-AD	Amyloid load (microPET)	Cohen et al., 2013
5XFAD	Florbetapir, PIB, and FDG PET	Rojas et al., 2013; Spencer et al., 2013
	Relaxation time changes (MRI)	
PS2APP	Vascular changes (ASL, VSI)	Weidensteiner et al., 2009
tTA/APP	Brain volumetry (3D MRI)	Badea et al., 2012
APPPS1 (APPPS1-21)	Plaque imaging (multiphoton *in vivo* imaging)	Hefendehl et al., 2011
	Targeting vascular cell adhesion molecule-1 expression (MRI)	Montagne et al., 2012
APPxPS1-Ki (APP^SL^PS1KI)	Perfusion (ASL)	Faure et al., 2011

## Visualizing vascular vs. parenchymal amyloid depositions

The amyloid hypothesis proposes that AD is caused by an imbalance between Aβ production and clearance which leads to parenchymal and vascular Aβ deposits (Hardy and Selkoe, 2002). Visualizing Aβ deposition in general is desirable to characterize the dynamics of this process and for testing of Aβ-directed therapeutics. To investigate the role of CAA in AD requires discriminating vascular from parenchymal Aβ deposits. Differentiation of the two compartments would enable monitoring the effects of Aβ removal strategies, which have been shown in some instances to increase CAA (Wilcock et al., 2004a, 2011). To date, assessment of Aβ load and CAA requires time-consuming postmortem neuropathological analysis. Imaging approaches enabling to assess Aβ deposition at the microscopic and macroscopic scale *in situ* are therefore welcome. The sub-micrometer spatial resolution of *in vivo* microscopic techniques allows differentiating CAA from neuritic amyloid deposits in a straightforward manner based on their spatial distribution within tissue. In contrast, monitoring vascular amyloid deposition using non-invasive macroscopic imaging with voxel dimensions of ≥50 μm is hampered by the fact that spatial resolution does not allow discriminating between the parenchymal and vascular compartments. Instead plaque subtype specific labeling is required, which remains a major challenge, given the chemical and structural similarity of the amyloid deposits. As addressed next, *in vivo* microscopy has evolved as an indispensable tool for studying the dynamics of CAA under experimental conditions and also for the development of amyloid subtype specific probes, which can then be appropriately labeled for macroscopic imaging investigations with e.g., optical techniques or PET.

The dynamics of CAA has been studied in real time in Tg2576 mice using multiphoton microscopy through cranial windows (Robbins et al., 2006). Affected vessels were labeled by methoxy-X04. Earliest appearance of CAA was observed as multifocal deposits of band-like Aβ in leptomeningeal arteries at approximately 9 months of age. Serial imaging sessions enabled monitoring growth of these deposits as well as appearance of new bands. The CAA progression in Tg2576 mice was found to be linear in the range of 9–16 months of age (Robbins et al., 2006). In contrast, APPswe/PS1dE9 mice showed CAA deposition in leptomeningeal arteries by 6 months of age (Garcia-Alloza et al., 2006). However, compared to Tg2576 animals, CAA progressed at a lower rate in these mice, which may be accounted for an increase of the Aβ_42_/Aβ_40_ ratio in APPswe/PS1dE9 mice.

Amyloid specific dyes such as Thioflavin, Congo red or curcumin have been used for the histopathological assessment of cerebral amyloidosis and CAA. Chemical modifications of these dyes have led to the development of specific imaging probes which can be employed to detect amyloid deposition *in vivo*. Alternative approaches explore the use of antibodies or antibody fragments for targeting vascular Aβ deposition. For this purpose, Aβ targeted compounds can be labeled with radionuclides such as ^11^C and ^18^F, fluorescent dyes or paramagnetic nanoparticles. However, the delivery of intravenously injected compounds can be affected by the status of the BBB as an impairment of the BBB function may lead to unspecific leakage of the probe. Moreover, species specific differences in the affinity sites of Aβ exist (Klunk et al., 2005), which in some cases does not allow for simple translation of approaches targeting Aβ across different species.

**Figure 2:**
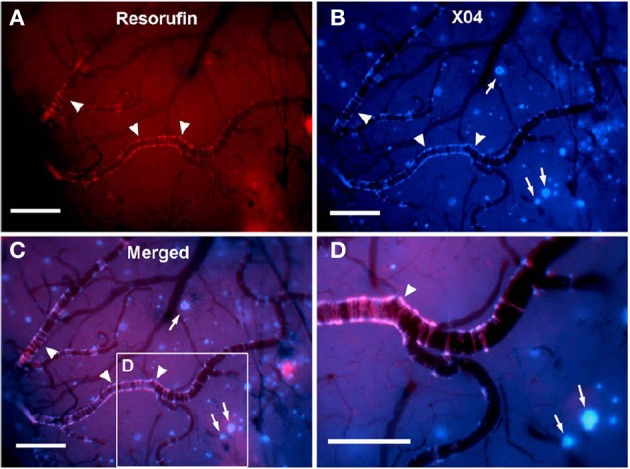
***In vivo* live imaging of CAA amyloid deposits through cranial window**. Closed cranial windows were prepared on the right parietal bone of 16-month-old Tg2576 mice and the congophilic amyloid binding dye, methoxy-X04 (X04), was administered (6 mg/kg i.p.). On the next day, 2 μM resorufin (dissolved in artificial CSF) was superfused over the brain through a closed cranial window for 5 min. After washing with artificial CSF for 10 min, live fluorescent images of resorufin (red) and X04 (blue) were taken. **(A)** Intense fluorescent labeling detected within the walls of the leptomeningeal arteries (arrowheads) but not in neuritic plaques after topical application of resorufin. **(B)** In contrast, topical application of methoxy-X04 labeled Aβ aggregates in both cerebral arteries (arrowheads) and parenchymal neuritic plaques (arrows). **(C)** Resorufin- and X04-images merged. **(D)** Magnified detail of **(C)**. Scale bars: 100 μm. Reproduced with permission from Han et al. (2011), © 2011 Han et al.

**Figure 3:**
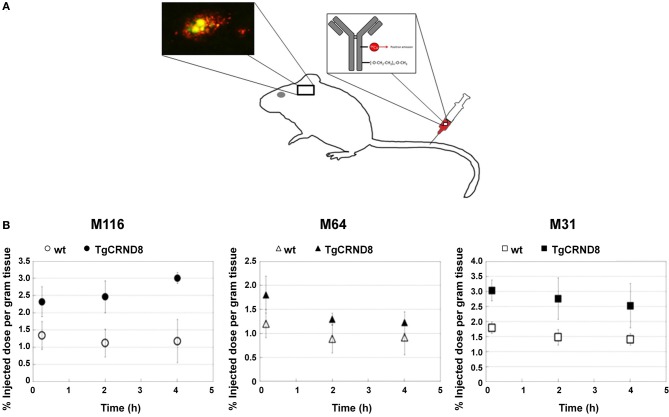
**Targeting specific of Aβ with PET compatible radiolabelled antibodies in the brains of living mice**. **(A)** Antibodies offer an opportunity to image specific types of Aβ pathology because of their excellent specificity. In the TgCRND8 mouse model of AD, two antibodies, M64 and M116, that target parenchyma aggregated Aβ plaques and one antibody, M31, that targets vascular Aβ were tested. All three antibodies were administered i.v. after labeling with both poly(ethylene glycol) (PEG) to enhance circulation and ^64^Cu to allow PET detection. **(B)** Quantitation of PET images (% of injected dose per gram tissue) in the brain 5 min, 2 h, and 4 h after i.v. of the probes: M116 showed progressive accumulation of M116 in the TgCRND8 brain vs. a lower, constant amount in the wild-type brain; M64 showed no difference in accumulation between TgCRND8 and wild-type mice at any time point; M31 showed greater accumulation in TgCRND8 mice than wild-type, but at a constant amount. Modified with permission from McLean et al. (2013), © 2013 American Chemical Society.

Established PET tracers such as the [^11^C]-Pittsburgh compound B or [^18^F]-florbetapir enable cerebral Aβ detection (Johnson et al., 2007; Wong et al., 2010), but do not allow the differentiation of neuritic plaques from CAA. Thus, a new series of [^18^F]-styrylpyridine derivatives has been developed which showed labeling of vascular Aβ in *in vitro* autoradiography of brain sections of patients with CAA or AD (Zha et al., 2011). Fluorescent dyes with specificity for vascular Aβ have also been synthesized. For example, Han et al. (2011) found that the phenoxazine derivative resorufin binds preferentially to vascular amyloid deposits as compared to neuritic plaques in aged Tg2576 transgenic mice, in contrast to methoxy-X04 which binds to both (Figure 2). Along the same lines, McLean et al. (2013) developed a method to translate a panel of anti-Aβ antibodies, which show excellent histological properties, into live animal imaging contrast agents. The antibodies M116 and M64 targeting neuritic plaques and M31 binding to vascular Aβ were labeled with ^64^Cu and injected into TgCRND8 mice. M31 and M116 were found to be significantly retained in the brains of transgenic mice after intravenous injection, while M64 was not (Figure 3). Immunohistological examination confirmed the specificity of the antibodies for either vascular or parenchymal Aβ deposits. Similarly, Nabuurs et al. (2012) investigated the properties of two heavy chain antibody fragments, ni3A and pa2H (Harmsen and De Haard, 2007; Rutgers et al., 2009), which in APP/PS1 mice showed affinity for neuritic plaques and CAA, in contrast to observations in human tissue, where ni3A was found to specifically target vascular Aβ. An antibody-based approach for MRI detection was developed by Poduslo et al. (2011). The monoclonal antibody, IgG4.1, was labeled with monocrystalline iron oxide nanoparticles. These conjugated nanoparticles bound to vascular amyloid deposits in arterioles of Tg2576 mice after infusion into the external carotid artery. The selectivity of the nanoparticle approach was fostered by the fact that the nanoparticles cannot cross the BBB and thus remained in the vascular compartment.

In summary, several studies have successfully demonstrated that CAA can be visualized in transgenic mice *in vivo* using different targeting strategies. While microscopic techniques are invasive and therefore confined to yield mechanistic information in animals, they constitute an important complement to macroscopic imaging approaches like PET and MRI which can also be used in humans. These imaging assays could be used in the future to address how vasculopathy is temporally linked to vascular Aβ deposition, but also how risk factors of AD, for example hypertension, affects this process. Moreover, the tools might be useful to evaluate Aβ removal strategies.

## Imaging cerebral amyloid clearance

It has been implicated that Aβ accumulation in the brain is not only the result of faulty Aβ production but also of an impaired Aβ clearance (Bell and Zlokovic, 2009). Mechanisms of cerebral Aβ clearing include degradation by proteases, interstitial fluid drainage, and transport of Aβ across the BBB (Weller, 1998; Deane et al., 2004, 2008). As discussed in the present section, imaging approaches have revealed aberrant vascular clearance mechanisms in transgenic models of AD.

Arbel-Ornath et al. (2013) have used multi-photon microscopy to visualize interstitial fluid drainage along perivascular spaces in APPswe/PS1dE9 in real time. The kinetics of dye clearance was studied after parenchymal dye injections in transgenic mice and wildtype controls 2.5–3 and 6–8 months of age. A significant impairment of the interstitial fluid drainage was observed in the old transgenic mice compared to young transgenic mice and age-matched wildtype mice.

Moreover, it has been shown that Aβ is a substrate for efflux transporters, enabling to traffick Aβ across the BBB (Kuhnke et al., 2007). Imaging strategies have been developed to visualize efflux transporter function by quantifying the uptake of substrates of these transporters. For example, (R)-[^11^C]-verapamil has been developed as a PET tracer to study P-glycoprotein function (van Assema et al., 2012). Higher (R)-[^11^C]-verapamil binding potential values were observed in AD patients compared to healthy controls, indicative of a decreased P-glycoprotein function.

**Figure 4:**
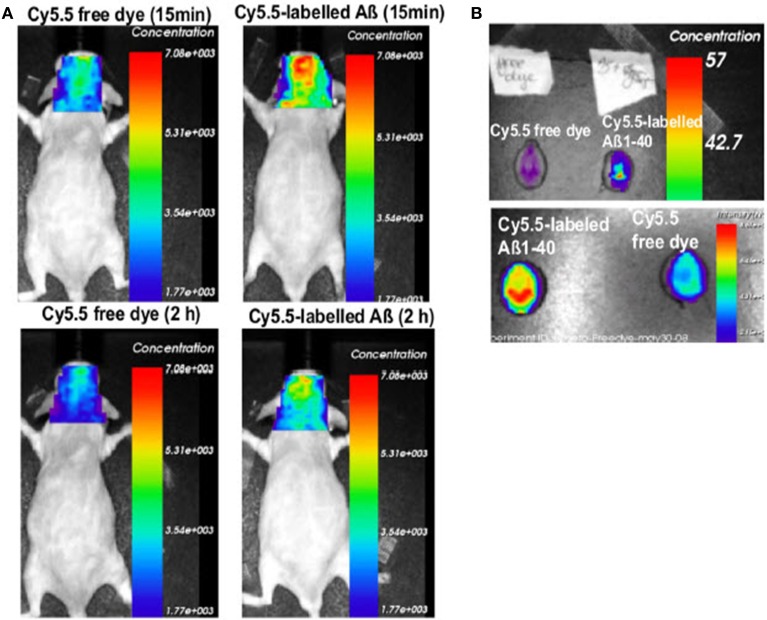
**Absence of Abcg2 allows more A β peptides to be transported into the brain**. **(A)** Two pairs of Abcg2 knockout mice were injected i.v. Cy5.5-free dye or Cy5.5-labeled Aβ_1 − 40_ peptides in equal fluorescence intensity. Animals were scanned alive using a NIRF imager at 15 min and 2 h. **(B)** NIRF scans of *ex vivo* brains collected at the end of the experiment. Signal intensity was significantly higher in the brains of Abcg knockout mice injected with Cy5.5-labeled Aβ peptides compared with Cy5.5 free dye (*t*-test, *p* < 0.001). This demonstrates that Cy5.5 was brought into the brain as a form of Cy5.5-labeled Aβ_1 − 40_ peptide, indicating that Abcg2 is required at the BBB to prevent the entry into the brain of circulating Aβ peptides. Modified with permission from Xiong et al. (2009), © 2009 Society for Neuroscience.

In a different approach the role of the drug efflux transporter ABCG2 was studied in a transgenic mouse model. ABCG2 is a 72 kDa transmembrane protein that forms functional homodimers and operates as BBB drug efflux transporter (Doyle and Ross, 2003). It has been shown that this transporter is significantly upregulated in AD/CAA brains at both the mRNA and protein levels (Zhang et al., 2003). It has been shown that ABCG2 is also increased in Tg-SwDI and 3XTg-AD mouse models (Xiong et al., 2009). The role of ABCG2 in Aβ transport at the BBB was investigated by Xiong et al. (2009) in Abcg2-null and wildtype mice after intravenous injection of Cy5.5-labeled Aβ_1–40_ or scrambled Aβ_1–40_. NIRF imaging of live animals showed that Abcg2-null mice accumulated significantly more Aβ in their brains than wildtype mice (Figure 4), a finding that was confirmed by immunohistochemistry. These results suggest that ABCG2 may act as a gatekeeper at the BBB to prevent blood Aβ from entering into the brain.

Taken together, these imaging studies provide a mechanistic link between cerebrovascular disease and AD where an impaired Aβ clearance promotes further amyloid deposition. If a defective clearance might constitute an initiating event for Aβ deposition needs to be investigated, but should be considered a new target for therapy in AD and CAA.

## Detection of chronic cerebral hypoperfusion

This section is devoted to studies comprising the use of microscopic or macroscopic imaging techniques to assess alterations in hemodynamic function due to deposition of Aβ in and around vessels as well as to changes in vasoactive mediators. For example, Dorr et al. (2012) observed a prolonged transit time of a fluorescent dye bolus in TgCRND8 mice compared to wildtype littermates using two-photon microscopy. Assessment of hemodynamic parameters covering the whole brain can be made with MRI. For dynamic susceptibility contrast MRI (DSC-MRI), T_2_- or T^*^_2_-weighted images are acquired serially. Regional changes in MRI signal intensity are measured as the contrast agent traverses the cerebral vasculature during its first-pass following intravenous bolus injection (Villringer et al., 1998). This information is then converted into contrast-time curves. The intravascular indicator dilution theory has been used to derive the hemodynamic parameters mean transient time, cerebral blood volume (CBV) and flow (CBF). Determination of absolute hemodynamic parameters requires calibration of the perfusion maps by the arterial input function (Rausch et al., 2000). Moreover, the theory assumes that the contrast agent remains intravascular during its passage. This is often not the case under pathological conditions where the BBB function may be compromised, thus leading to leakage of the injected tracer. Modeling of the leakage contribution to the image signal intensity changes has been used to obtain information on the vascular transfer constant, i.e., BBB permeability (Johnson et al., 2004). Instead of introducing an exogenous label, moving blood can also be labeled magnetically. These MRI methods are based on arterial water as a freely diffusible tracer (Williams et al., 1992). For arterial spin labeling (ASL) a non-equilibrium state (typically spin inversion) is generated to tag inflowing spins at a level proximal to the imaging slab. Images are recorded following a transit delay to allow these tagged spins to enter the imaging plane and exchange with tissue. Control images are required to compensate for direct saturation effects (Williams et al., 1992). Quantitative CBF values can be obtained from ASL images.

**Figure 5:**
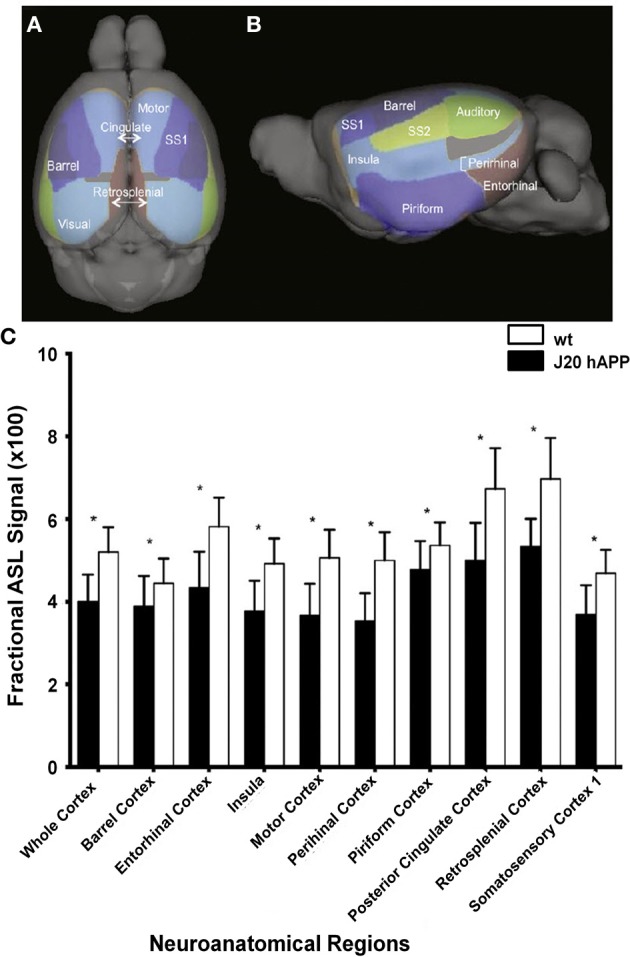
**Hypoperfusion in 3-month-old J20 hAPP mice modeling AD**. Superior **(A)** and lateral **(B)** views of the cortical surface atlas with 14 regions-of-interest labels derived from high resolution 3D MRI data sets. **(C)** ASL perfusion MRI measurements from representative regions-of-interest in young transgenic and age-matched wild-type mice. Note that the whole cortex and most regions demonstrated significantly lower perfusion (^*^p < 0.05) in J20 hAPP compared with wild-type animals. Modified with permission from Hébert et al. (2013), © 2013 Elsevier Inc.

Cerebral hypoperfusion has been observed in AD patients using MRI (Johnson et al., 2005; Chen et al., 2011a), and has been suggested to be an early biomarker for the disease (Alsop et al., 2010; Chao et al., 2010). However, the mechanism underlying the perfusion deficits are poorly understood (Chen et al., 2011a). A decreased metabolic demand (Chen et al., 2011b) and decreased microvascular density (Buee et al., 1994) have been suggested as plausible causes, however, studies linking directly perfusion with pathological and molecular *postmortem* read-outs have not been attempted in humans and might be difficult to achieve. Studies assessing impairment of hemodynamic function in mice overexpressing APP can be pivotal in this regard, as a correlation of imaging studies with postmortem analysis of brain tissue can be conveniently performed. In several studies ASL was applied to APP mouse strains which have only sporadic CAA. A significant reduction in CBF has been observed in the occipital cortex of 10- to 17-month-old PS2APP (Weidensteiner et al., 2009), in 6-month-old APPxPS1-Ki (Faure et al., 2011), in 12-month old APP/PS1 (Poisnel et al., 2012), in 3-, 12- and 18-month-old J20 hAPP (Hébert et al., 2013) (Figure 5), and in 12- to 16-month-old Tg2576 mice (Massaad et al., 2010) compared to the respective age-matched controls. Perfusion was normal in subcortical (thalamic) areas in the transgenic mice (Faure et al., 2011; Poisnel et al., 2012). Reduced CBV levels at rest were also observed in the cerebral cortex, hippocampus, and thalamus of PDAPP mice compared to wildtype controls, while values were similar in other brain regions (Wu et al., 2004). In contrast, Hooijmans et al. (2007a) reported that CBF was not significantly reduced in 18-month-old APP/PS1 mice when performing bolus tracking of D_2_O using deuterium MRS.

The question which needs to be addressed is why these mice show reduced cerebral perfusion. A histopathological study has shown a decreased capillary density around senile Aβ plaques (Koutnetsova et al., 2006) which might explain the perfusion deficit, but areas of decreased perfusion did not correlate with plaque load (Weidensteiner et al., 2009). Another possibility is that reduced CBF may be due to lower cerebral metabolic demand of the brain tissue in APP mice. However, a study comparing cerebral glucose metabolism as assessed with [^18^F]-fluoro-2-deoxy-D-glucose PET and ASL-derived perfusion showed no correlation between the two read-outs (Poisnel et al., 2012). Cerebral glucose uptake decreased in the hippocampus, cortex and striatum of 3-month-old APP/PS1 mice, but increased in these brain regions in 12-month-old mice at an age when CBF is compromised, thus suggesting alternative mechanisms. Several studies using transgenic APP mice demonstrated alterations in vasoactive signaling (Niwa et al., 2001) and in the renin-angiotensin system (Takeda et al., 2009), as well as the generation of reactive oxygen species (Iadecola et al., 1999; Tong et al., 2005) in the brains of APP mice, all of which can directly affect vascular tone. Impairment of vascular function is observed in APP overexpressing mice prior the onset of plaque deposition and appears to be mediated by soluble Aβ (Han et al., 2008; Park et al., 2013). Indeed hypoperfusion was observed in mouse strains at this young age (Faure et al., 2011; Hébert et al., 2013).

Vascular deposition of Aβ is not a prerequisite for vascular dysfunction in AD, but CAA aggravates the functional deficit (Park et al., 2013). Aβ can exert direct vascular effects by attenuating the endothelium-dependent vasodilation (Paris et al., 2000, 2003; Luo et al., 2008), triggering the production of reactive oxygen species (Tong et al., 2005; Park et al., 2008; Massaad et al., 2010) and inducing remodeling of the vessel wall (Merlini et al., 2011). In addition, vascular accumulation of Aβ has been associated with the deposition of fibrin, which can lead to vessel stenosis and occlusion (Paul et al., 2007; Cortes-Canteli et al., 2010; Klohs et al., 2012). Cerebral hypoperfusion accelerates CAA (Okamoto et al., 2012), induces oxidative stress and alterations of the renin-angiotensin system (Washida et al., 2010), and may thus initiate a vicious cycle. Moreover, it has been shown that transgenic mice overexpressing APP have an impaired cerebral autoregulation (Niwa et al., 2002). The disability of the cerebral vasculature in the presence of Aβ to respond to changes in perfusion pressure constitutes another mechanism of vascular pathology in AD.

Hypoperfusion seems to be a critical process in the pathogenesis of AD and further investigation into its mechanism is warranted for developing therapeutic interventions that can abrogate the functional deficits. MRI has been demonstrated to be a robust technique to assess perfusion in large areas of the human and small animal brain, with or without administration of contrast agent. To obtain information at a higher spatial resolution, laser Doppler flowmetry or two-photon microscopy may be applied. But for this, cranial windows are necessary, and the information is obviously limited to upper cortical regions.

## Alteration of stimulus evoked response in functional imaging—changed neurovascular coupling or impaired neuronal function?

Functional imaging read-outs may constitute early sensitive markers of underlying pathology, since alterations in neuronal function and vascular reactivity are expected to precede any gross changes in anatomy as detected with structural imaging techniques. One caveat for functional imaging studies are that they are routinely performed in anesthetized animals. As the anesthetic may affect neuronal activation and/or neurovascular coupling and thus have an effect on hemodynamic read-outs (Masamoto and Kanno, 2012), the protocol needs to be carefully controlled. It is discussed next how imaging based on CBF and CBV read-outs are suitable to conduct functional imaging studies in order to investigate changes in these parameters in response to neuronal activation in transgenic animals modeling AD.

Two-photon imaging has been shown to be a unique approach to studying vascular dysfunction in mouse models of AD, by evaluating neurovascular function e.g., through analyses of functional hyperemia evoked by sensory stimulation. Using this technique, Takano et al. (2007) demonstrated *in vivo* that reactive changes of astrocytes and abnormalities of the microcirculation occur in early stages of the disease preceding amyloid deposition and neuronal loss. In contrast to the low Ca^2+^ signaling activity in non-stimulated control animals, astrocytes in 2–4-month-old Tg2576 mice exhibited a higher frequency of spontaneous Ca^2+^ oscillations. Animals with abnormal astrocytic activity also displayed instability of the vascular tone with oscillatory cycles of relaxation/constriction of small arteries. Aβ administration increased the frequency of spontaneous astrocytic Ca^2+^ increases. Because astrocytes control local microcirculation and contribute to functional hyperemia (Anderson and Nedergaard, 2003; Takano et al., 2006), abnormal astrocytic activity may contribute to vascular instability in AD and thereby to compromised neuronal function.

Dorr et al. (2012) observed a prolongation of bolus transit times of a fluorescent dye in TgCRND8 mice during hypercapnia using two-photon microscopy. While in wildtype mice, due to CO_2_-induced vessel dilatation, the hypercapnic challenge led to a reduction of transit time as compared to animals breathing air, the opposite effect has been observed in transgenic animals. Also in the transgenic group there was an increase in transit time with age, i.e., with more severe Aβ pathology. It was concluded that this paradoxical response to hypercapnia resulted from compromised CO_2_-induced dilatation of the feeding arteries/arterioles in the presence of preserved venous dilatation and reflected a profoundly impaired vascular function in TgCRND8 mice.

Functional MRI (fMRI) comprises a number of techniques to non-invasively study brain function in humans and animals. In addition to CBV and CBF read-outs, a blood oxygenation level dependent (BOLD) contrast can be used for fMRI (Ogawa et al., 1992). fMRI can be performed at rest (Jonckers et al., 2011) or with different types of physiological stimuli like sensory, thermal, or electrical stimulation (Mueggler et al., 2003; Bosshard et al., 2010). Moreover, pharmacological fMRI can measure the hemodynamic responses induced by central nervous system active drugs or vasoactive compounds and can thus be used as surrogate reflecting the effects of these drugs on neural transmission and/or vessel function.

**Figure 6:**
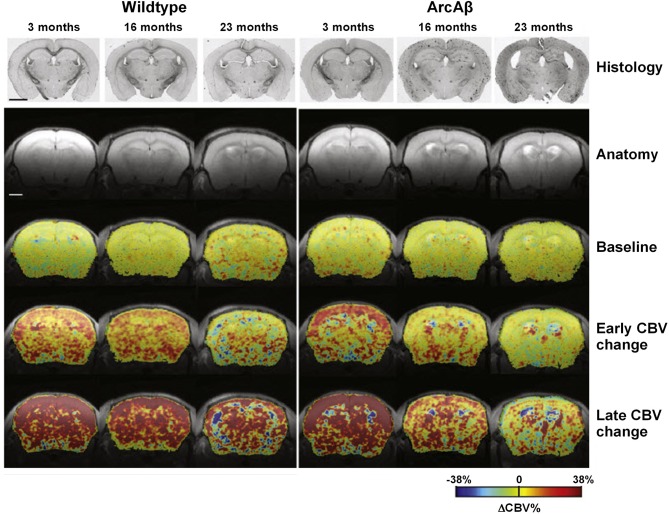
**Vascular response to acetazolamide decreased as a function of age in the arcA β mouse model of cerebral amyloidosis, exemplified in color-coded MRI-derived CBV maps**. Images for a representative age-matched wild-type control littermate and an arcAβ mouse of each age group. Histological sections stained for Aβ amyloid deposition as well as anatomical MR reference images are displayed in the two top rows. Histology reveals Aβ deposition in 16- and 23-month-old but not 3-month-old arcAβ mice, while none of the wild-type animals displayed any amyloid pathology. The color-coded CBV maps superimposed on the anatomical scans represent baseline ΔCBV% values, early changes in ΔCBV% and maximum ΔCBV% values (ΔCBV%, max). The early ΔCBV% response in arcAβ mice decreased significantly as a function of age as compared to age-matched wild-type mice. Similarly ΔCBV%, max significantly decreased in arcAβ mice as a function of age. In 3-month-old animals no difference between wild-type and arcAβ mice has been found in either parameter. The scale bar represents 2 mm. Reproduced with permission from Princz-Kranz et al. (2010), © 2010 Elsevier Inc.

APP23 mice of various ages have been analyzed using fMRI (Mueggler et al., 2002, 2003). CBV changes were detected in 6-, 13–15- and 25-month-old mutant mice in response to pharmacological stimulation using the GABA_A_ receptor antagonist, bicuculline, physiological stimulation by inducing hypercapnia using the carbonic anhydrase inhibitor, acetazolamide, and peripheral sensory activation using electrical stimulation of the hind paws. In 13–15- and 25-month-old APP23 mice, all three stimulation paradigms evoked CBV responses that were significantly smaller when compared to age-matched, control littermates (Mueggler et al., 2002, 2003). In young animals of 6 months of age, there was no difference between the transgenic and wildtype group. Princz-Kranz et al. (2010) demonstrated a diminuished CBV response upon stimulation with acetazolamide in the cortex of 16- and 23-month-old arcAβ mice compared to age-matched wildtype littermates, while there was no difference between 3-month-old ArcAβ mice and controls (Figure 6). Both the rate of vascular adaptation (vascular reactivity) and the extent of the dilatation (as a measure for the reserve capacity) were found to be impaired in aged ArcAβ mice.

A challenge in fMRI is the interpretation in animal models of AD. Under physiological conditions neurovascular coupling is rather tight (Logothetis et al., 2001; Schulz et al., 2012), but under pathological conditions a reduced fMRI response may indicate either a decrease in neuronal activity, an impaired neurovascular coupling or both. Sanganahalli et al. (2013) have shown in a non-transgenic rat model without CAA that the cortical BOLD response and neuronal activity upon sensory stimulation are reduced in rats with inducible amyloid pathology while the neurovascular coupling remains unaffected. But neurovascular coupling may be impacted in the presence of CAA as Aβ exerts direct vascular effects. Luo et al. (2008) intravenously injected Aβ_1–40_ in anesthetized C57BL/6 mice.Injection of the peptide led to a significant reduction in CBV in a dose-dependent and region-specific manner while the injection of phosphate buffered solution or of the reversed peptide, Aβ_40–1_, did not induce any significant change in vascular response. This vasconstrictive effect might also explain the impaired vascular reactivity in mice with CAA upon acetazolamide and hypercapnia challenge (Mueggler et al., 2003; Princz-Kranz et al., 2010; Dorr et al., 2012). Given the attractiveness of performing fMRI also in AD patients, further studies are warranted to examine how changes in neurovascular mediators impact fMRI read-outs.

## Visualizing amyloid-induced vascular remodeling

Changes in hemodynamics of the vasculature will inevitably lead to vascular remodeling. In patients, Aβ deposits are seen in leptomeningeal and cortical arteries, and less frequently in veins and capillaries (Buee et al., 1994; Thal et al., 2008a,b). Transgenic mice show a larger heterogeneity of phenotypes with capillaries and large arteries affected by Aβ deposition. Imaging approaches targeting the vasculature at a phenotypic level are attractive tools to study remodeling of the vascular architecture as a consequence of CAA. The use of MRI to detect vascular remodeling in transgenic models is discussed in this section.

Magnetic resonance angiography (MRA) comprises a number of MRI techniques capable of visualizing the vascular architecture non-invasively. The method has limited spatial resolution, but is translational and routinely used in the clinics. Time-of-flight MRA (TOF-MRA) generates contrast between signals arising from stationary tissue and flowing blood. Maximum intensity projection or volume-rendered visualization delivers 3D representations of the cerebral vasculature in humans (Talagala et al., 1995) and rodents (Beckmann et al., 1999; Reese et al., 1999; Beckmann, 2000). However, the technique is inherently dependent on the orientation of the blood vessels with respect to the imaging plane and the actual flow velocity of the blood (Lin et al., 1997; Reese et al., 1999). While TOF-MRA can depict major brain arteries, parts of the vasculature such as small intracortical arteries, which branch off the larger cerebral vessels, and veins displaying slower blood flow velocities than arteries, are difficult to be visualized. The quality of TOF-MR angiograms is governed by the vascular anatomy and the blood flow characteristics. Signal voids in TOF-MRA may indicate absence of flow, low flow velocity, or turbulent flow. Microturbulences for instance translate into MRA signal voids due to the loss of signal coherence despite the fact that the vessel is still fully perfused (Krucker et al., 2004). Nevertheless, the degree of vasculopathy may be graded based on number and extent of signal voids detected on the angiograms in a semiquantitative manner (El Tayara et al., 2010; Kara et al., 2012). In contrast-enhanced MRA (CE-MRA) a paramagnetic contrast agent is intravenously administered, which causes a signal loss due to increased signal dephasing (El Tayara et al., 2010; Klohs et al., 2012). The CE-MRA data image can be used like in TOF-MRA data to visualize the 3D vessel architecture. However, in CE-MRA flow and motion artifacts are smaller compared to TOF-MRA (Mellin et al., 1994; Lin et al., 1997).

**Figure 7:**
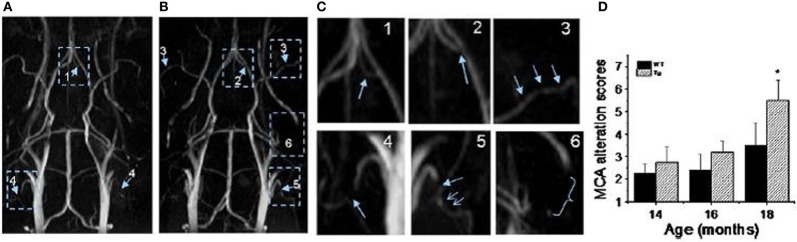
**MR angiography of transgenic mice modeling AD**. **(A,B)** MR angiograms of 18-month-old Tg2576 mice collected at 17.6 T showing various levels of severities of morphological changes appointed in 3D maximum intensity projection. The number indicates the appointed score to the level of severity of alterations. For example: 1, a flow disturbance (as seen in anterior communicating artery in image **A**); 2, a small signal void (as observed at the origin of anterior communicating artery in image **B**); 3, more than two small voids in same artery (as observed on the middle cerebral artery (MCA) on both sides in image **B**); 4, an extended void (as observed in the external carotid artery on both sides in image **A**); 5, a combination of an extended void and several small signal voids (as observed in the external carotid artery on both sides in image **B**); 6, the signal is no longer visible (as shown at the pterygo portion of the pterygopalatine artery in image **A**,**B**). The enlarge view of alterations is shown in **(C)**. **(D)** MCA alteration mean score in control and Tg2576 mice with age. Values are mean ± SE (error bars); one-tail student *t*-test; ^*^P < 0.05; *n* = 4. Reproduced with permission from Kara et al. (2012), © 2011 Elsevier Inc.

TOF-MRA has been applied to probe vascular remodeling in APP23 mice *in vivo* (Beckmann et al., 2003). Flow voids were detected at the internal carotid artery of 11-month-old APP23 mice. At the age of 20 months, additional flow disturbances were observed in the circle of Willis. Vascular corrosion casts (Krucker et al., 2004; Meyer et al., 2008) obtained from the same mice revealed that vessel elimination, deformation, or both had taken place at the sites where flow voids were detected by TOF-MRA. The detailed 3D architecture of the vasculature visible in the casts assisted the identification of smaller vessels most likely formed as substitution or anastomosis within the Circle of Willis. Thal et al. (2009) observed blood flow disturbances in TOF-MR angiograms in 25- to 26-month-old APP23 mice which corresponded to CAA-related capillary occlusion in the branches of the thalamoperforating arteries as seen with histology. El Tayara et al. (2010) evaluated vascular alterations in APP/PS1 and in PS1 mice. The double transgenic model is relatively aggressive as extracellular amyloid deposition starts at the age of 2.5 months (Blanchard et al., 2003). However, unlike plaque deposition, severity of cerebrovascular alterations is stabilized in older animals. Alterations of the middle cerebral artery were detected in old APP/PS1 mice by evaluating the severity of signal voids and the reduction of patent length of the vessel using TOF-MRA and CE-MRA. MRA obtained at very high magnetic fields (17.6 T) improved the capability to visualize smaller vessels (Kara et al., 2012). Visual and quantitative analysis of angiograms revealed severe blood flow defects in large and medium sized arteries in Tg2576 mice (Figure 7). In particular blood flow defects were observed in the middle cerebral and in the anterior communicating artery in Tg2576 mice. Histological data show that Aβ deposits in the vessel wall may be responsible for impaired CBF.

The use of cryogenic radiofrequency probes improves the quality of mouse brain angiograms at lower magnetic fields (Baltes et al., 2009). Klohs et al. (2012) employed this technology to quantitatively assess age-dependent changes of the cortical vasculature in the ArcAβ model of cerebral amyloidosis. To estimate the density of the cortical microvasculature *in vivo*, CE-MRA was used, based on the acquisition of data before and after administration of superparamagnetic iron oxide (SPIO) nanoparticles allowing the visualization of intracortical microvessels with high-resolution. A significant reduction in the number of functional vessels (radii of 20–80 μm) has been observed in 24-month-old ArcAβ mice compared with age-matched wildtype mice, whereas there was no difference between transgenic and wildtype mice at 4 months of age. Immunohistochemistry demonstrated strong fibrinogen and Aβ deposition in small- and medium-sized vessels, but not in large cerebral arteries, of 24-month-old ArcAβ mice. The reduced density of transcortical functional vessels may thus be attributed to vascular occlusion caused by deposition of Aβ and fibrin, which translated into impaired perfusion. Fibrin deposition has been observed previously in TgCRND8 mice (Paul et al., 2007; Cortes-Canteli et al., 2010) and since fibrin-binding probes are currently under development (Starmans et al., 2013) it may become possible to visualize cerebral fibrin deposition in these transgenic models *in vivo* in the near future.

The microvasculature which includes capillaries cannot be visualized directly with current MRA techniques. For this purpose, methods have been developed based on measuring the changes in the relaxation rates R^*^_2_ and R_2_ after administration of a paramagnetic contrast agent with long blood half-life. Relaxation has been exploited in vessel size imaging, where maps can provide insight into the composition of vessel sizes in the brain *in vivo* (Tropres et al., 2001). A method closely related is to measure the relaxation rate shift index Q (Jensen and Chandra, 2000), where the index is sensitive to the density but not the size of microvessels. Weidensteiner et al. (2009) determined vessel size and density in different brain regions in PS2APP mice but observed no significant differences to wildtype littermates. However, in this strain CAA is sparse and affects only large arteries.

MRI has rendered itself the most versatile methodology to visualize vascular networks of large regions or even of the whole brain while retaining a sufficient high resolution to assess smaller vessels and to provide an estimation of microvascular density. Despite the fact that in transgenic mouse models it has been shown that CAA affects the cerebral vasculature at different hierarchical levels, what causes such structural alterations in cerebral vessels is not yet known. Chronic changes in levels of vasoactive mediators like soluble Aβ, vascular endothelial growth factor, transforming growth factor-1, and altered signaling or density of vascular receptors might be implicated and future imaging studies might address this by visualizing vascular remodeling in models where these medidators are modified. Moreover, imaging studies might be useful to elucidate the role of risk factors of AD like diabetes and hypertension on the vasculature of the AD brain. Indeed, hypertension, atherosclerosis, diabetes, dyslipidemia and adiposity may impact on vascular structure and function to promote neurodegenerative processes and instigate AD (see Kalaria et al., 2012 for a recent review). The presence of vascular pathology involving arterial stiffness, arteriolosclerosis, endothelial degeneration and BBB dysfunction leads to chronic cerebral hypoperfusion, which in turn induces several features of AD pathology including selective brain atrophy, white matter changes and accumulation of abnormal proteins such Aβ. To our knowledge, no imaging studies addressing specific questions related to atherosclerosis in AD mouse models have been reported so far. Nevertheless, it is worth stressing the fact that very important developments have been achieved in molecular imaging of atherosclerosis (the interested reader is referred to reviews by Lobatto et al., 2011; Owen et al., 2011). Linking plaque anatomy and function to inflammation may help considerably to elucidate the mechanisms and complications related to atherosclerosis in AD.

## Targeting neurovascular inflammation

A hallmark of AD is neuroinflammation which has been implicated to drive and even trigger neurodegeneration (Krstic and Knuesel, 2013). Inflammation involves also the cerebral vasculature, though the role of inflammation in the vasculopathy is not well understood. Macrophages and microglia surround amyloid affected vessels (Maat-Schieman et al., 1997; Vinters et al., 1998) and circulating macrophages have been shown to migrate from the lumen into the vessel wall (Vinters et al., 1998). Intercellular adhesion molecule-1 is upregulated at the endothelium in the AD brain (Frohman et al., 1991). Moreover, the inflammatory response of the vasculature is increased in the presence of Aβ (Vromman et al., 2013). A few imaging studies suggesting that inflammation might have deleterious consequences on vascular function are discussed next.

Different strategies to image vascular inflammation have been pursued comprising the labeling of inflammatory cells, the use of fluorogenic substrates for enzymes and fluorescent or PET probes targeted against inflammatory receptors (Wunder and Klohs, 2008; Wunder et al., 2009; Aalto et al., 2011; Li et al., 2013). Garcia-Alloza et al. (2009) have observed a strong association between CAA, matrix metalloproteinases and oxidative stress in leptomeningeal vessels of APPswe/PS1dE9 and Tg2576 with multiphoton microscopy and fluorogenic probes. The matrix metalloproteinases activity was found to be associated with matrix degradation and loss of vascular integrity.

**Figure 8:**
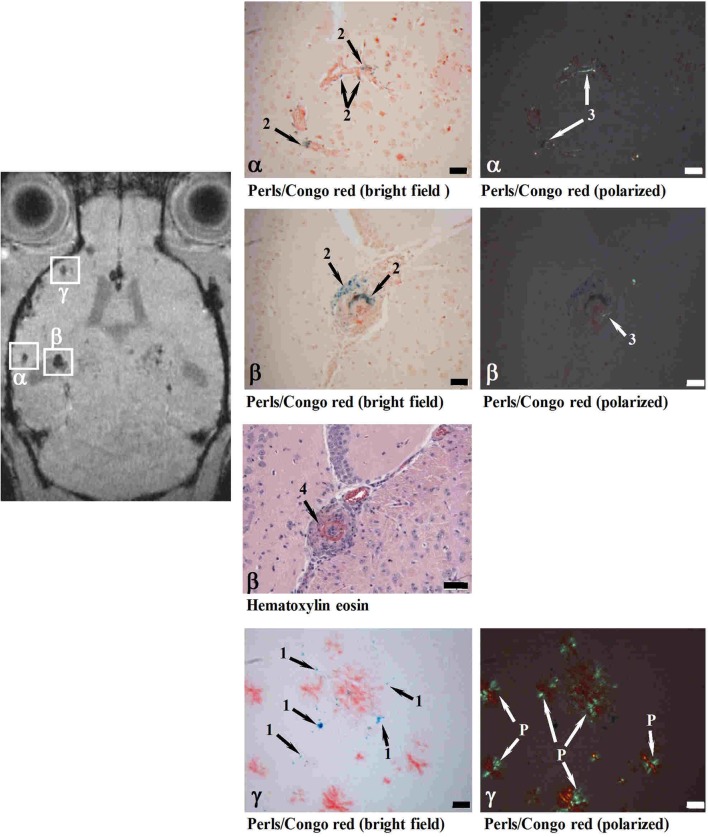
**MRI detection of CAA-related microvascular alterations utilizing superparamagnetic iron oxide (SPIO) particles**. Histological examination of cerebral cortex sites with foci of attenuated MRI signal (α, β, γ). At 24 h following SPIO administration, a male 28-month-old APP23 mouse was analyzed *in vivo* by MRI and processed for histology immediately thereafter. Perls/Prussian blue staining showed iron-loaded macrophages in CAA-laden vessels (Congo red positive) at both sites (α and β). While the vessel walls were thickened at both α and β locations, only site β showed in addition vasculitis characterized by lymphocyte infiltration (Hematoxylin eosin). At site γ, isolated iron-loaded macrophages were present close to amyloid vessels. 1, Iron in isolated macrophages; 2, iron in macrophages at the vessel wall; 3, amyloid deposit in vessel wall; 4, vasculitis; P, amyloid plaque. Scale bars, 50 μm. Congo red-stained sections were observed under bright field or polarized light. Reproduced from Beckmann et al. (2011), © 2011 the authors.

In a different approach, mice of different transgenic lines have been examined with MRI following the intravenous administration of SPIO nanoparticles (Beckmann et al., 2011), which were hypothesized to having been taken up by circulating monocytes through absorptive endocytosis (Weissleder et al., 1990; Beckmann et al., 2009). Foci of signal attenuation were detected in cortical and thalamic brain regions of aged APP23 mice (Figure 8). Histology confirmed the presence of iron-containing macrophages in the vicinity of CAA-affected blood vessels, suggesting that the foci of signal attenuation detected *in vivo* might be associated with CAA in the transgenic model. A fraction of the sites additionally showed thickened vessel walls and vasculitis. Consistent with the visualization of CAA-associated lesions, MRI detected a much smaller number of attenuated signal sites in APP23xPS45, APP24, and APP51 mice, which develop significantly less CAA and microvascular pathology than APP23. These results are consistent with monocytes and microglia being involved in amyloid deposition in the wall of capillaries and in perivascular plaques (Wegiel et al., 2004). Montagne et al. (2012) have used an antibody targeting the vascular adhesion molecule-1 (VCAM-1) coupled to microparticles of iron oxide (MPIO). After injection of the probe MRI showed a significantly higher number of signal voids in the brains of 20-month-old APP/PS1 compared to age-matched wildtype controls. Immunohistochemistry revealed that VCAM-1 was overexpressed in APP/PS1 mice in all the brain regions studied (cortex, hippocampus and cerebellum). Despite APP/PS1 mice develop only minimal amyloid angiopathy (Radde et al., 2006) a significant cerebrovascular inflammation was detected in the cerebellum of these animals, which was associated with intravascular Aβ deposition (Montagne et al., 2012). Interestingly, the expression of VCAM-1 was significantly higher in the cerebellum compared to the cortex in transgenic mice. Accordingly, signal voids induced by MPIOs-αVCAM-1 and detected by MRI were significantly increased in APP/PS1 mice in all structures compared to age-matched wildtype mice.

Microglial-vascular interactions may play a critical role in the amplification and perpetuation of inflammatory reactivity in AD brain. Indeed, *post-mortem* examination of medial temporal cortical tissue from humans revealed that microgliosis was progressively increased from non-demented controls to mild AD to severe AD with the latter demonstrating areas of clustered microglia (Jantaratnotai et al., 2010). Microglial clusters in severe AD brain were in close proximity with extravascular laminin and also plasma protein, fibrinogen, implicating vascular perturbation as a component of inflammatory reactivity. Microscopy studies of microglial function in murine AD models may help to better understand microglial-vascular interactions.

So far high resolution *in vivo* studies of microglial function were conducted in mice with genetically labeled microglia. However, because of the low expression levels of green fluorescent protein, some mouse lines are less suitable for studying the role of microglia under pathological conditions. The availability of a non-genetically encoded, easy to use marker, enabling high quality staining of microglia in any mouse strain at any experimental age would obviously be very attractive. Schwendele et al. (2012) utilized tomato lectin from *Lycopersicon esculentum* (Acarin et al., 1994; Boucsein et al., 2000) for high resolution *in vivo* imaging of microglia. A brief pressure injection of tomato lectin conjugated with a fluorescent dye (DyLight® 594) into the mouse cortex resulted in robust staining of microglial cells and blood vessels. The latter were easily distinguished from microglia based on their morphological appearance. The reliability of the *in vivo* staining protocol was tested in different mouse lines.

Since vascular inflammation has been implicated to partake in the deleterious consequences of CAA like degeneration of vascular smooth muscle cells and hemorrhage (Maat-Schieman et al., 1997), but still very little is known between the interaction of inflammation and vascular pathology. Further studies are warranted to investigate when and how inflammation is involved.

## Detection of blood-brain barrier integrity loss and of cerebral microbleeds

Severe CAA is characterized by the degeneration of the vessel wall, leading to a double-barreled appearance of the vessels with an intact adventitia, a thickened basement membrane that contains Aβ-deposits, and a widely degenerated smooth muscle cell layer (Thal et al., 2008a). Areas of fibrinoid necrosis can be frequently observed in these vessels. Degeneration of vascular smooth muscle cells lead to a loss of BBB function and eventually to vessel rupture with the occurrence of CMBs and hemorrhage. In this section, we address the detection of BBB leakage and of CMBs using MRI.

Imaging of the BBB with MRI has been widely applied to pathologies such as brain tumors and metastases, stroke and head trauma (Giesel et al., 2010). In dynamic contrast-enhanced MRI (DCE-MRI), a series of images is acquired during intravenous bolus injection of Gd-based contrast agents. Kinetic modeling of the contrast agent can provide information on vascular leakage (Tofts and Kernode, 1991). The method has for example been used to predict the occurrence of hemorrhage after ischemic stroke (Kassner et al., 2005). Klohs et al. (2013) have performed a longitudinal MRI study where DCE-MRI was applied in ArcAβ and wildtype mice. While vascular leakage of the contrast agent was significantly associated with age, there was no effect of genotype. This finding was surprising as compromised BBB function has been described for the ArcAβ strain (Merlini et al., 2011). When aged ArcAβ mice were injected intravenously with Trypan blue, leakage of the dye was observed around Aβ-affected vessels. Moreover, aged ArcAβ mice showed CMBs indicative of severe vascular pathology (Klohs et al., 2011, 2013).

The observation made in the transgenic animals is in line with studies in AD and MCI patients, where no differences in contrast agent kinetics have been detected with respect to healthy controls (Caserta et al., 1998; Starr et al., 2009). The discrepancy of the DCE-MRI findings might be explained by the fact that BBB dysfunction in AD is subtle and diffuse when compared to diseases such as brain tumors, multiple sclerosis and stroke, for which the impairment is relatively large and focal (Giesel et al., 2010). Hence, DCE-MRI may not be sensitive enough for detecting BBB impairment in mouse models of AD *in vivo*.

**Figure 9:**
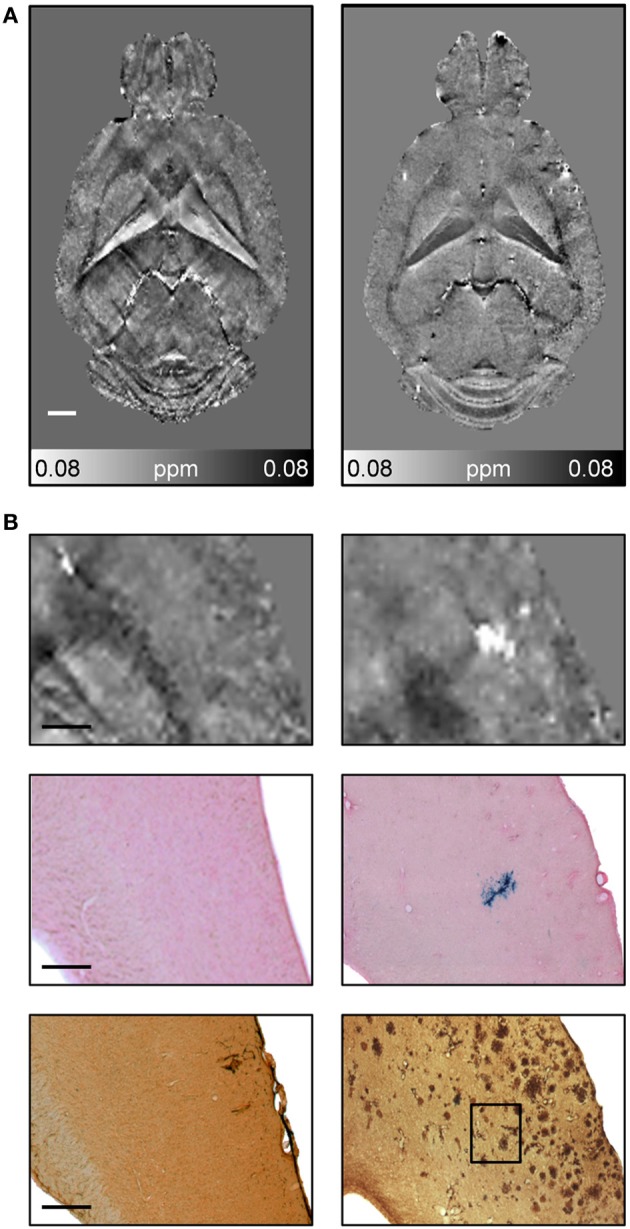
**Detection of CMBs using quantitative susceptibility mapping MRI**. **(A)** Horizontal quantitative susceptibility maps of an 18-month-old wild type animal and of an age-matched transgenic arcAβ mouse. **(B)** Quantitative susceptibility maps with corresponding tissue section after Prussian blue/eosin staining and anti-Aβ immunohistochemistry. Focal areas of high susceptibility in the cortex of 18-month old arcAβ mice correspond to areas of focal iron accumulation, indicating the occurrence of cerebral microbleeds in this mouse strain. Modified from Klohs et al. (2011), © 2011 ISCBFM.

CMBs and hemorrhages can be detected with CT and MRI techniques with the latter being more often used for diagnosis (Sperling et al., 2011). T^*^_2_-weighted gradient-echo MRI protocols, which are sensitive to paramagnetic iron compounds such as hemosiderin found in blood degradation products, reveal CMBs in patients as focal hypointensities typically occurring as round or ovoid areas (Pettersen et al., 2008; Ayaz et al., 2010; Sperling et al., 2011). The occurrence of CMBs has also been reported for transgenic APP mice with CAA. For example, Beckmann et al. (2011) described the presence of foci of low signal intensity in cortical and thalamic brain regions of aged APP23 mice. Klohs et al. (2011) demonstrated in ArcAβ mice that quantitative susceptibility mapping provides increased detection sensitivity of CMBs and improved contrast when compared with conventional T^*^_2_-weighted gradient-echo magnitude imaging (Figure 9). Quantitative susceptibility maps were generated from phase data acquired with a high-resolution T^*^_2_-weighted gradient-echo sequence depicting both the localization and spatial extent of CMBs with high accuracy.

Taken together, assessment of BBB with current DCE-MRI methods does not seem to be sensitive enough to detect vascular leakage. Advances in imaging technology enable the improved diagnostic detection of CMBs in patients and animal models. The assessment of CMB load can be used in studies to estimate the severity of CAA and to monitor the effect of therapy.

## Assessing the effects of therapies targeting vascular pathology in AD

Clinical therapeutic trials in AD patients performed so far were disappointing. Based on activities in animal models suggesting that prevention or early intervention may be a viable strategy for AD treatment, there is a trend toward treating patients at very early stages of disease or even preventatively (Bateman et al., 2012; Fleisher et al., 2012). Obviously, biomarker development, including imaging, is an essential part of this endeavor, in order to select the right patients to be treated early (Reiman et al., 2012). In this section, we briefly discuss a few therapy-intervention studies in animals addressing vascular pathology related to AD.

Therapeutic strategies have targeted APP processing, as well as the trafficking of soluble Aβ and strategies to remove aggregated Aβ. Gregory et al. (2012) analyzed in Tg2576 mice Aβ deposition in vessels and clearance from vascular walls and their relationship to the concentration of Aβ in the brain. Levels of Aβ in the brain were modulated either by peripheral clearance through administration of gelsolin which binds with high affinity to plasma Aβ (Matsuoka et al., 2003), or by directly inhibiting its formation via administration of LY-411575, a small-molecule γ-secretase inhibitor. Both gelsolin and LY-411575 reduced the rate of CAA progression in Tg2576 mice in the absence of an immune response. The progression of CAA was also halted when gelsolin was combined with LY-411575. These data suggest that CAA progression can be prevented with non-immune therapy approaches that may reduce the availability of soluble Aβ. Yet there was no evidence for substantial clearance of Aβ already deposited at vessels.

Yang et al. (2011) assessed the therapeutic potential of blocking apolipoprotein E (ApoE)/Aβ interactions, by administering an Aβ fragment (Aβ_12–28P_) to young TgSwDI mice (from 3 to 9 months of age). Increased cognitive function, decreased cortical, hippocampal and thalamic fibrillar vascular amyloid burden, and decreased extent of cerebral hemorrhages was found in treated compared with untreated TgSwDI mice. While this therapeutic strategy holds promise in young mice, it would be of interest to verify whether it would be effective in older animals.

**Figure 10:**
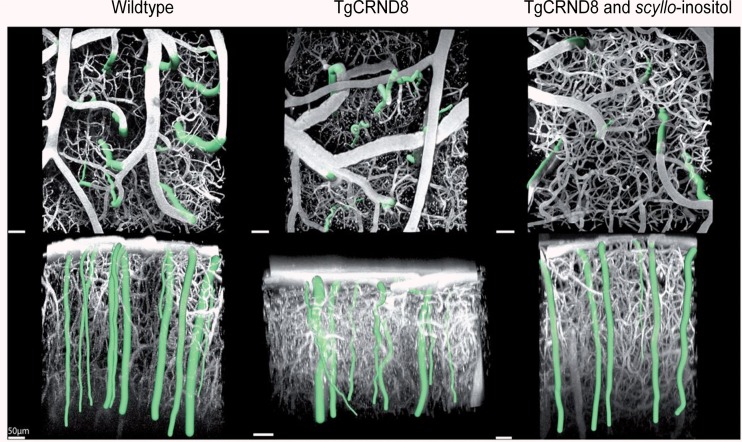
**Segmentation of cortical penetrating vessels overlaid on maximum intensity projections of cortical microvasculature obtained from *in vivo* two-photon fluorescence microscopy images of the cortical microcirculation of 6.5–12-month-old mice: parallel to cortical surface (top row) and perpendicular to cortical surface (bottom row)**. Penetrating vessels for each individual mouse are highlighted. Average tortuosity of penetrating vessels for each sample subject (mean ± standard error): wild-type mice 1.03 ± 0.003, transgenic TgCRND8 mice 1.10 ± 0.006, *scyllo*-inositol-treated TgCRND8 mice 1.03 ± 0.005. Reproduced with permission from Dorr et al. (2012), © 2012 the authors.

Two studies demonstrated the stereoisomer of inositol, *scyllo*-inositol, to have potential therapeutic properties to treat CAA. When given orally to TgCRND8 mice, *scyllo*-inositol inhibited Aβ aggregation into high-molecular-weight oligomers in the brain and ameliorated several AD-like phenotypes in these mice, including impaired cognition, altered synaptic physiology, and cerebral Aβ pathology (McLaurin et al., 2006). These therapeutic effects, which occurred regardless of whether the compound was given before or well after the onset of the AD-like phenotypes, support the idea that the accumulation of Aβ oligomers plays a central role in the disease pathogenesis. Multiphoton laser scanning microscopy examinations of TgCRND8 mice *in vivo* revealed that structural changes of cortical arterioles (increase in tortuosity and decrease in caliber) with amyloid-β peptide accumulation were accompanied by progressive functional compromise, reflected in higher dispersion of microvascular network transit times, elongation of the transit times, and impaired microvascular reactivity to hypercapnia in the transgenic mice (Dorr et al., 2012). However, administration of *scyllo*-inositol rescued both structural and functional impairment of the cortical microvasculature (Figure 10). Overall, these results suggest microvascular impairment to be directly correlated with Aβ accumulation, highlighting the importance of targeting CAA clearance for effective diagnosis, monitoring of disease progression and treatment of AD.

Epidemiological studies have provided evidence that statins, cholesterol-lowering drugs broadly used in the treatment of cardiovascular diseases, have therapeutic potential in AD (Jick et al., 2000; Wolozin et al., 2000) and to a slower cognitive decline in mild-to-moderate AD patients (Sparks et al., 2006). Studies in animals have revealed that the effects of statin treatment are not due to their vascular and anti-inflammatory effects rather than their cholesterol-lowering effect. Tong et al. (2012) reported that a 3–6 months treatment with simvastatin completely rescued cerebrovascular reactivity, basal endothelial nitric oxide synthesis, and activity-induced neurometabolic and neurovascular coupling in adult (6 months) and aged (12 months) J20 hAPP transgenic mice. Remarkably, simvastatin fully restored short- and long-term memory in adult mice, but not in aged AD mice. These beneficial effects occurred without any decreasing effect of simvastatin on brain Aβ levels or plaque load. However, in AD mice with recovered memory, protein levels of the learning- and memory-related immediate early genes c-Fos and Egr-1 were normalized or upregulated in hippocampal CA1 neurons, indicative of restored neuronal function. Simvastatin also restored the CBF response in the somatosenory cortex to whisker simulation in 6- and 12-month-old J20 hAPP mice, and restored whisker-stimulated cerebral glucose uptake in the somatosensory cortex of 12-month-old APP mice as assessed by FDG-PET. These findings disclose new sites of action for statins against Aβ-induced neuronal and cerebrovascular deficits that could be predictive of therapeutic benefit in AD patients. They further indicate that simvastatin and, possibly, other brain penetrant statins bear high therapeutic promise in early AD and in patients with vascular diseases who are at risk of developing AD.

Treatment with angiotensin receptor blockers has been associated to reduce AD-related pathology (Hajjar et al., 2012) or with a lower risk and slower disease progression compared to other antihypertensive agents (Li et al., 2010). These data indicate that angiotensin receptor blockers not only decrease blood pressure but also decrease vascular inflammation may effectively reduce the risk of developing AD (Hajjar et al., 2012). Wang et al. (2007) screened 55 clinically prescribed antihypertensive medications for AD-modifying activity using primary cortico-hippocampal neuron cultures generated from Tg2576 mice. Despite 7 antihypertensive agents reduced Aβ accumulation, only valsartan was capable of attenuating oligomerization of Aβ peptides into high-molecular-weight oligomeric peptides, known to be involved in cognitive deterioration. Preventive treatment of Tg2576 mice with valsartan significantly reduced AD-type neuropathology and the content of soluble extracellular oligomeric Aβ peptides in the brain. Most importantly, valsartan administration also attenuated the development of Aβ-mediated cognitive deterioration. These preclinical studies suggest that certain antihypertensive drugs may have AD-modifying activity and may protect against progressive Aβ-related memory deficits in subjects with AD or in those at high risk of developing AD.

Aβ removing therapies are currently tested in clinical trials and have also been studied in transgenic animals, yielding conflicting results. Using multiphoton microscopy, Prada et al. (2007) showed that anti-Aβ passive immunotherapy can remove cerebral Aβ in Tg2576 mice, depending on the duration of treatment. Clearance of CAA and neuritic deposits was detected within 1 week after a single administration of 10D5, an antibody against the N-terminal of Aβ, directly to the brain, but the effect on CAA was only transient. Moreover, the progression rate of CAA became greater in the antibody treated group, suggesting that vascular Aβ deposition may accelerate after short-lived clearance. Chronic administration of the antibody over 2 weeks led to a more robust clearance of CAA. Other studies have shown that Aβ immunotherapy may also at least transiently worsen CAA, with increased incidence of cerebral microhemorrhages in aged transgenic mice (Bard et al., 2000; Pfeifer et al., 2002; Wilcock et al., 2006; Schroeter et al., 2008; Thakker et al., 2009). This is in line with a study where SPIO-enhanced MRI revealed a higher number of sites with signal attenuation in APP23 mice following a chronic treatment with the Aβ antibody β 1 (Beckmann et al., 2011). Histological analyses demonstrated an increased number of CAA vessels and of iron loaded macrophages in the vicinity of CAA vessels, for mice receiving the β 1 antibody. In addition, a study using T^*^_2_-weighted MRI for the detection of CMBs in Tg2576 mice treated with either a non-selective antibody (6G1) targeting soluble and insoluble Aβ or a more selective antibody (8F5) targeting primarily soluble Aβ (Luo et al., 2010). Both antibodies increased CMB incidence in aged APP transgenic mice compared with baseline or vehicle treatment.

**Figure 11:**
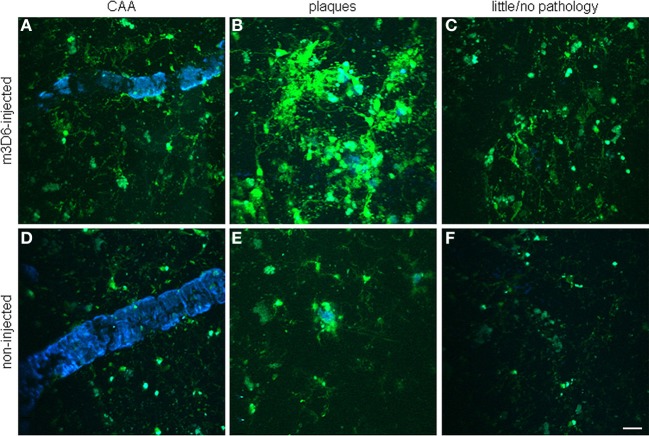
**Rapid microglial response around amyloid pathology after systemic anti-A**β **antibody administration in PDAPP mice**. Peripheral m3D6 administration results in marked morphological changes in microglia. Three-dimensional reconstructed z-series stack two-photon microscopy images taken of 22-month-old PDAPP±;CX3CR1/green fluorescent protein± mice injected with 500 μ g of m3D6 **(A–C)**, an anti-Aβ antibody, or not injected **(D–F)**. Green fluorescent protein-labeled microglia are green. Fibrillar amyloid was labeled with methoxy-XO4 (blue). Scale bar, 20 μm. Reproduced with permission from Koenigsknecht-Talboo et al. (2008), © 2008 Society for Neuroscience.

It has been hypothesized that the antibodies exert their beneficial as well as their deleterious effects via an antibody Fc domain-mediated microglial activation and Aβ phagocytosis (Bard et al., 2000; Wilcock et al., 2004b). Koenigsknecht-Talboo et al. (2008) demonstrated that the effects of antibodies that recognize aggregated Aβ are rapid and involve microglia. The anti-Aβ antibody, m3D6, that binds to aggregated Aβ (Cirrito et al., 2003), was administered to PDAPP mice, an AD mouse model that was bred to contain fluorescent microglia. Three days after systemic administration of m3D6, there was a marked increase in both the number of microglial cells and processes per cell visualized *in vivo* by multiphoton microscopy (Figure 11). These changes required the Fc domain of m3D6 and were not observed with mHJ5.1, an antibody specific to soluble Aβ.

Taken together, these studies demonstrate the potential of animal studies in therapy studies. Imaging studies are expected to play a pivotal role in this regard; their application ranging from safety testing of putative drugs e.g., detection of microbleeds, to elucidating mechanism of action, to monitoring of treatment efficacy.

## Translating imaging findings from animal models of AD

Translational research from animal models to clinical studies and from human studies back to animal models is relevant for the development and validation of imaging biomarkers of AD. In particular, MRI as well as PET assays are well suited for translational applications. While many imaging findings presented here, for example chronic cerebral hypoperfusion or the occurrence of CMBs, have been observed both in transgenic mice overexpressing APP and in AD patients, some studies have reported a lack of concordance between imaging findings in mice and men. For example, the uptake of an amyloid PET tracer reflected the amount of Aβ in AD patients but not in transgenic mice due to species-specific differences in the affinity sites of Aβ between mice and humans (Klunk et al., 2005). In another example, Luo et al. (2012) observed a glucose hypermetabolism in the brain of 7-month-old Tg2576 mice, despite the fact that hemodynamic read-outs were not different to wildtype mice. This is in contrast to AD patients, where glucose hypometabolism and hypoperfusion were concomitantly observed (Nagata et al., 2000). The increase in glucose metabolism in Tg2576 has been attributed to a neuronal compensatory mechanism due to the APP overexpression and might thus constitute an artifact of the model.

In general, there are limitations in employing models for developing biomarkers of AD. Transgenic strains overexpressing APP have been widely used because they reproduce essential histopathological features and molecular mechanisms of AD. But these models might resemble more the familial forms of AD (<1% of AD cases) or Down syndrome rather than the sporadic, late-onset form of AD. Some double and triple transgenic lines have been generated to overlay amyloid with tau and presenilin pathology (Blanchard et al., 2003; Oddo et al., 2003a,b). But even these strains do not recapitulate the complex pathophysiology of sporadic AD, which affects most AD patients. Moreover, while it is advantageous from an experimental perspective that the disease pathology in animals develops quickly, it could be an important feature of human AD that converging mechanisms which contribute to the impairment of brain function occur over a very long span and might be modulated by life-style choices and comorbidies.

Additional challenges occur when developing new therapies. Main potential reasons for the lack of concordance between preclinical models and human clinical trials could be wrong targets, incomplete models, lack of individual variability in the animal models, patients enrolled too late and comorbidities. As patients participating in clinical trials are heterogenous whereas most models utilize in-bred mouse strains, evaluating novel treatments in multiple lines may help to address this point. Also, the lack of substantial cell loss in the majority of rodent models may indicate that they better represent some aspects of the prodromal phase of the disease.

Emerging studies foster the relationship between vascular disease and tauopathy in AD. Recently, several *post mortem* studies on the brains of AD patients provided evidence that cardiovascular pathology like atherosclerosis is highly correlated with neuritic plaques, tau neurofibrillary tangles, and CAA (Roher et al., 2003; Beach et al., 2007; Yarchoan et al., 2012). However, imaging approaches addressing vascular aspects have not been applied to transgenic tau models so far. Similarly to the use of probes for targeting amyloid, such studies may profit from the recent development of PET tracer to image tau (Okamura et al., 2012; Maruyama et al., 2013).

Finally, animal models displaying chronic hypoperfusion (Shibata et al., 2004) or multiple microinfarcts (Wang et al., 2012b) and spontaneous hypertensive rats (Calcinaghi et al., 2013) are being used to investigate the role of vascular risk factors and pathological mechanisms in the etiology of dementia.

## Conclusion

The potential of imaging techniques to study cerebrovascular pathology in animal models of AD has just been started to be fully exploited. The presented examples using genetically engineered mice demonstrate the extreme versatility of the application of imaging tools, which can provide information on gross vascular morphology and probe hemodynamic function down to address the basic mechanisms underlying CAA and neurovascular dysfunction. While the microscopic techniques are inherently invasive and thus not translatable to AD patients, the non-invasive techniques like PET and MRI can be translated and might thus provide biomarkers of the disease. Imaging of animal models is ideally suited for developing such diagnostic assays. But challenges remain in the selection of a suitable animal model to address a specific research question related to the vascular aspects of AD.

### Conflict of interest statement

Nicolau Beckmann and Derya R. Shimshek are employees of Novartis Pharma AG, Basel, Switzerland. The other authors declare that the research was conducted in the absence of any commercial or financial relationships that could be construed as a potential conflict of interest.
